# Microbiota and the clock: sexual dimorphism matters!

**DOI:** 10.18632/aging.102051

**Published:** 2019-06-20

**Authors:** Benjamin D. Weger, Frédéric Gachon

**Affiliations:** 1 Institute of Bioengineering, School of Life Sciences, Ecole Polytechnique Fédérale de Lausanne, Lausanne CH-1015, Switzerland; 2 Institute for Molecular Bioscience, The University of Queensland, St. Lucia, QLD 4072, Australia

**Keywords:** circadian clock, microbiota, sexual dimorphism, growth hormone

Obesity-associated metabolic disorders represent a major problem for public health. Alteration of both the circadian clock and microbiota have been implicated with the development of such metabolic disorders. The circadian clock is an endogenous 24-hour oscillator that is present in virtually every cell of an organism helping it to adapt to daily environmental changes. Microbiota are an agglomerate of bacteria and microorganism cohabitating with its host. Scientists have observed over the past decade that microbiota have a strong impact on host physiology. Many metabolic pathways which are affected by microbiota are also controlled by the circadian clock. This observation suggests that these two systems might cooperate to regulate host physiology. Indeed, an interaction between microbiota and the circadian clock has been reported recently (reviewed in [1]). The abundance of certain bacteria in gut microbiota are rhythmically oscillating only in the presence of a functional host circadian clock, which seems to be mainly driven by clock-controlled feeding rhythms. Conversely, there is increasing evidence indicating that microbiota host interactions are not a one-way street and host physiology can be influenced by microbiota. However, there were contradictory reports concerning the role of the microbiome on the host circadian clock in peripheral tissues. It was, thus, unclear to what extent and how microbiota and the circadian clock cooperate to impact diurnal host physiology.

Our recent work has transcriptionally and metabolically profiled several metabolic organs of germ-free mice around the course of a day [2]. The acquired data revealed that the circadian core clock in these organs stays broadly unaffected. However, rhythmic downstream metabolic pathways controlled by the circadian clock such as amino acid, lipid, steroid and drug metabolism were affected when microbiota were absent. Moreover, these pathways are reported to be different between females and males (i.e. sexually dimorphic). Indeed, we observed that germ-free male mice appeared feminized in their gene expression and metabolism. Conversely, germ-free females were masculinized. Although it is well known that metabolic organs such as the liver and white adipose tissue differ strongly between male and female, we were surprised to discover that microbiota are involved in the establishment of this sex difference. Mechanistically, we demonstrated that these phenomena are caused by altered Growth Hormone secretion and an associated defective sexual maturation in germ-free mice. Thus, both changes are tightly interconnected and responsible for the sexual differences of the liver. Additionally, the activation of Aryl Hydrocarbon Receptor (AHR) also seems to play a role in the altered sexual dimorphism. This is interesting as AHR is a well described xenobiotic receptor that can sense substances secreted by the microbiota and plays a role in innate immunity and sexual development [3,4].

Collectively, our study uncovers a new mechanism involving Growth Hormone signaling that microbiota utilizes to regulate the metabolism of their host. The described mechanism behind the feminization and masculinization can explain the previously reported differences in metabolism, xenobiotic detoxification, poor reproduction and the resistance to liver cancer in germ-free mice. In fact, these phenomena are at the same time hallmarks of feminized physiology of the germ-free male mice exclusively used in almost all these studies. Concordantly, patients with a Growth-Hormone receptor deficiency are resistant to diseases such as cancer and diabetes [5].

Moreover, differences between sexes are highly relevant for physiology and pathology. For example, women are less prone to develop liver cancer than men. On the other hand, after puberty women have a higher risk of developing Crohn’s disease, a chronic inflammatory disease of the gastrointestinal tract [6]. Interestingly, both pathologies are associated with altered intestinal microbiota [7,8]. As microbiota play a role in sustaining sexual differences, it might provide an alternative understanding of these differences mechanistically and its role in the pathophysiology of these diseases. Furthermore, future work will show if an infection or taking antibiotics during childhood (a treatment which reduces microbiota diversity) interfere with sexual maturation and, potentially, later pathogenesis in adulthood.
